# Ecotourism effects on health and immunity of Magellanic penguins at two reproductive colonies with disparate touristic regimes and population trends

**DOI:** 10.1093/conphys/coy060

**Published:** 2018-11-13

**Authors:** Maria G Palacios, Verónica L D’Amico, Marcelo Bertellotti

**Affiliations:** 1 Applied Ecophysiology Group, Centro para el Estudio de Sistemas Marinos, Consejo Nacional de Investigaciones Científicas y Técnicas (CESIMAR-CONICET), Boulevard 2915 (9120) Puerto Madryn, Chubut, Argentina; 2 Department of Production, Environment and Sustainable Development, University of Chubut, Alem 1573 (9120) Puerto Madryn, Chubut, Argentina

**Keywords:** Conservation physiology, ecoimmunology, haematology, stress, tourism, wildlife

## Abstract

Negative effects of ecotourism on wildlife are rising worldwide. Conservation physiology can play a major role in protecting wildlife by providing early alerts on changes in the status of individuals exposed to tourist activities. We measured an integrated set of immune and health-state indices to evaluate the effects of ecotourism on Magellanic penguins (*Spheniscus magellanicus*). We studied two reproductive colonies that differed in the intensity of tourism and population trends: Punta Tombo (higher tourism intensity, declining population) and San Lorenzo (lower tourism intensity, growing population). Within each colony, we compared individuals from an area that was exposed to tourists and a control area where tourism was excluded. Adult penguins exposed to tourism at Punta Tombo, but not at San Lorenzo, showed physiological alterations indicative of chronic stress (higher heterophil to lymphocyte ratios) and parasitic infection (elevated heterophil and eosinophil counts). Penguin chicks exposed to tourism at Punta Tombo, but not at San Lorenzo, also showed physiological alterations indicative of poor immune and general-health condition: lower humoral innate immunity, haematocrit, and glucose levels and higher inflammatory responses likely due to increased prevalence of fleas. Our results indicate that individuals of a declining population exposed to high levels of tourism express physiological indicators of chronic stress and poor health that could make adults and juveniles vulnerable to disease. These effects are expressed despite a long history of exposure and behavioural habituation to human visitation. In contrast, individuals of a growing population exposed to more recent and lower levels of tourism showed no effect. Our study demonstrates how a diverse physiological toolkit within a conservation physiology approach can provide important information for a better comprehension of anthropogenic effects on wild animals in our changing world.

## Teaser

Understanding the actual effects of ecotourism on wildlife is critical. Penguin adults and chicks exposed to higher-intensity ecotourism in a declining colony showed physiological indicators of chronic stress, altered immunity, and poor general health compared to non-visited within-colony controls and to individuals exposed to lower-intensity ecotourism in a growing colony.

## Introduction

Population declines and extinctions of wildlife are occurring globally as a consequence of human activity (Dirzo *et al.*, 2014; McCauley *et al.*, 2015). Increased awareness of the immediate importance of conserving natural ecosystems has led to increased attraction to visiting fragile, pristine natural areas, which exposes those communities to new pressures (Moorhouse *et al.*, 2015). Ecotourism, or nature-based tourism, is among the fastest growing human activities in many wilderness areas worldwide (Scheyvens, 1999). More than eight billion people visit natural protected areas per year globally (Balmford *et al.*, 2015) and numbers are predicted to keep increasing (Moorhouse *et al.*, 2015). Reports on negative effects of tourist activities on wild populations are rising, leading to growing concern about the actual effects of ecotourism on wildlife welfare and conservation, and the call to closely monitor populations subject to tourist activities (Geffroy *et al.*, 2015; Moorhouse *et al.*, 2015).

Conservation physiology can play a key role in this respect and physiological indicators are becoming increasingly important for determining anthropogenic effects on wild animals (Cooke *et al.*, 2013; Madliger *et al.*, 2016). A major advantage of physiological indicators is that they can provide an early alert on changes in the status of individuals, even before these adversely affect their reproduction and/or survival and, as a consequence, population dynamics (Carey, 2005; Wikelski and Cooke, 2006). Assessment of stress hormone levels has been a common and valuable tool in conservation physiology; however, a lack of consensus on the interpretation of observed patterns in wild animals (Madliger and Love, 2014) highlights the necessity of assessing other relevant physiological functions (e.g. immunological, haematological, nutritional, metabolic) in studies of anthropogenic effects on wildlife (Geffroy *et al.*, 2017; Madliger *et al.*, 2016).

**Figure 1:**
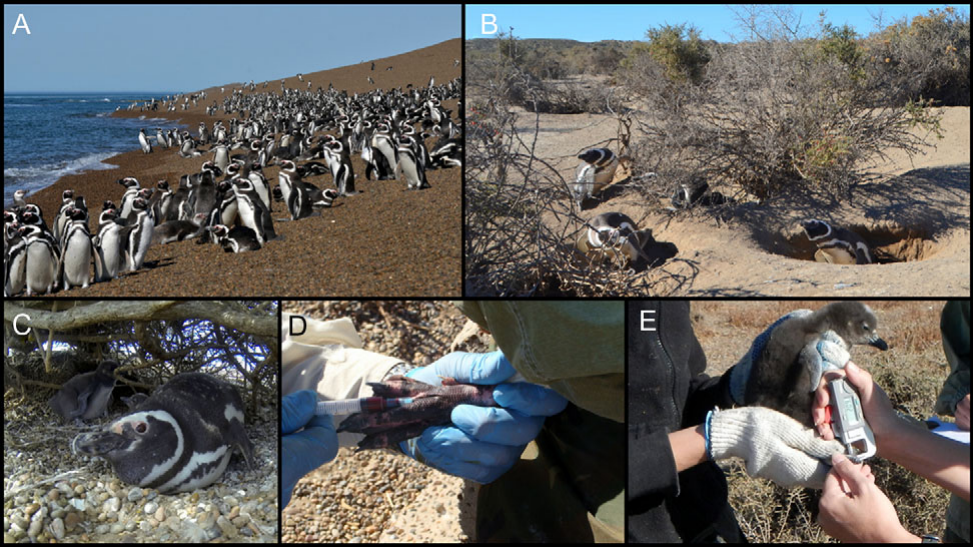
Magellanic penguins in northern Patagonia, Argentina. (A) Adults gathered at the shore. (B) Nesting grounds. (C) Adult and two chicks at a nest. (D) Blood collection from an adult. (E) Measuring the thickness of the interdigitary membrane of the foot of a chick during the PHA assay. Photo credits: Marcelo Bertellotti.

We present a study in Magellanic penguins *Spheniscus magellanicus* (Fig. 1) subject to ecotourist visitation that illustrates the advantages of a conservation physiology approach using a diverse physiological toolkit, reinforcing findings in other wildlife species (e.g. French *et al.*, 2010, marine iguanas; Semeniuk *et al.*, 2009, stingrays). Penguins constitute one of the most attractive and charismatic wildlife as a tourist resource (Boersma, 2008; Ellenberg, 2017). In contrast to other penguin species that show relatively high vulnerability to touristic activities (reviewed by Ellenberg, 2017), Magellanic penguins have generally shown low effect of tourism when assessed by behavioural and demographic indices (Villanueva *et al.*, 2012; Yorio and Boersma, 1992). Nevertheless, studies measuring stress hormone levels have yielded ambiguous results (Walker *et al.*, 2005, 2006), which have led to the dispute of the notion that Magellanic penguins are physiologically habituated to tourist visits and to the suggestion instead that tourist-exposed individuals might actually be chronically stressed (Cyr and Romero, 2009). Newborn chicks exposed to tourism in the largest colony of the species (Punta Tombo, Chubut, Argentina) mounted elevated corticosterone responses compared to chicks sampled at a control area within the colony (Walker *et al.*, 2005). In contrast, tourist-exposed adults showed depressed corticosterone responses due to decreased adrenal function compared to adults from the control area (Walker *et al.*, 2006). Whether such human-induced alterations in the stress responses of wild animals are beneficial or detrimental is presently unknown (Romero and Wikelski, 2002; Walker *et al.*, 2006; Cyr and Romero, 2009; Geffroy *et al.*, 2017). A much thorough understanding of the consequences of ecotourism and altered endocrine responses of wildlife induced by humans can be obtained using a broader battery of relevant physiological measurements (French *et al.*, 2010).

Here, we performed an integrated assessment of physiological functions measuring various immune and health-state indices to provide a more complete comprehension of the effects of ecotourism on Magellanic penguins. As documented in marine iguanas (French *et al.*, 2010), alterations of the endocrine stress axis of individuals exposed to ecotourism can result in depressed immunity and/or negative changes in their general-health status. Thus, following previous studies on the effects of tourism on penguins (e.g. Walker *et al.*, 2005, 2006; Villanueva *et al.*, 2012) and other wildlife (e.g. Amos *et al.*, 2006; French *et al.*, 2010), we compared physiological parameters of individuals exposed to tourist visitations to those of individuals not exposed to tourists in nearby locations within the same habitat. This approach minimizes potential dissimilarities in general environmental conditions (i.e. climate, landscape) individuals are exposed to, allowing a stronger test of the effect of tourism than comparisons among populations located in different, or more distant, habitats.

In contrast with the aforementioned findings in the reproductive colony at Punta Tombo, no effects of ecotourism on the stress response of Magellanic penguins have been detected in another colony of the species, the colony at San Lorenzo (Villanueva *et al.*, 2012). Compared to the colony at Punta Tombo, the colony at San Lorenzo has a more recent history of tourist visitation, lower intensity of annual tourist visits, fewer reproductive pairs, and an increasing population trend (Table 1). These factors, together with different tourist visitation practices (Supplementary information), might have contributed to the disparate effects of tourism on the stress responses of penguins between the two colonies (Villanueva *et al.*, 2012). Regardless of the specific underlying factors, which are presently unknown, the different stress patterns in response to tourism in the two reproductive colonies provide the opportunity to further evaluate the effects of tourist visitation on Magellanic penguins under contrasting contexts.

**Table 1:** Comparison of the two studied Magellanic penguin reproductive colonies

Colony	Punta Tombo	San Lorenzo
Number of breeding pairs	201 000^a^	135 000^b^
Age (years)	98	41
Years with tourism	>55	<20
Number of annual visitors	~100 000^c^	~10 000^d^
Population trend^b^	Declining^b^ (growth rate 0.99)	Increasing^b^ (growth rate 1.21)

^a^Rebstock *et al.* (2016).

^b^Pozzi *et al.* (2015).

^c^Villanueva *et al.* (2014).

^d^Secretaría de Turismo y Areas protegidas. Anuario Estadístico 2014/15.

Thus, in general, if tourist visitation has an impact on immunity and health of Magellanic penguins, we can expect tourist-exposed individuals to show depressed immunity and/or negative changes in their general-health status compared to their respective within-colony controls. In addition, adult males and females might differ in their response to environmental factors, including tourist visitation (e.g. Knapp *et al.*, 2013), thus, we sampled individuals of both genders. Similarly, mature and immature individuals could display different responses to tourist visitation, although this effect has seldom been assessed; therefore, for a more thorough evaluation we sampled both penguin adults and chicks. With respect to the two different colonies, we expected that the effects of tourism on immunity and health parameters might be evident, or more pronounced, in the colony where tourist-exposed penguins have previously shown altered endocrine stress responses (i.e. Punta Tombo) than in the colony where such alterations have not been found (i.e. San Lorenzo). Nevertheless, as the two colonies were sampled in separate years (see Methods), direct between-colony comparisons in physiological parameters was not performed.

## Materials and methods

### Study sites and sampling design

Magellanic penguins breed in reproductive colonies along the South American coast in Chile, Argentina, and the Malvinas (Falkland) Islands (Bertellotti, 2013; Boersma *et al.*, 2013).

**Figure 2:**
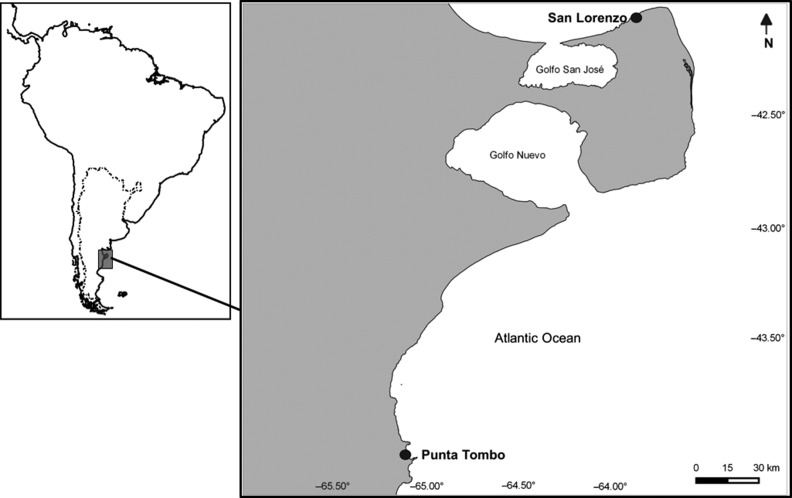
Location of the two studied Magellanic penguin reproductive colonies in the Atlantic coast of northern Patagonia, Argentina.

The colonies at Punta Tombo (44° 02′ S, 62° 11′ W) and San Lorenzo (42° 50′ S, 63° 49′ W) are located within Natural Protected Areas in the arid coast of Patagonia, Argentina (Fig. 2). Within each colony, we selected a control (i.e. non-visited) area located nearby (1–1.5 km), but out of sight from, the visited area. Except for the presence of walking trails, the two areas were comparable in terms of landscape features, nest types and density, breeding phenology, and distance to the shore. All selected nests in the tourist-visited area of each colony were within 15 m of walking trails, with most of them located within 5 m, and could be visually observed from the trails.

Penguins were sampled during early December, when adults were rearing young chicks. Sampling at San Lorenzo was performed during 3 days in 2013 (9, 10, and 16 December) and at Punta Tombo during 2 days in 2015 (6 and 7 December). Twenty nests having two chicks each were randomly selected in the tourist-visited and the non-visited area of each colony. Sampling of tourist-visited and control nests within each colony was balanced with respect to date and time of the day to minimize heterogeneity. The caring adult at the time of sampling and both chicks were removed from the nest and blood samples (0.5–2.5 ml) were obtained from the metatarsal vein using sterile heparinized syringes and sterile techniques. Time elapsed between initial nest disturbance and end of blood collection was recorded for each individual and evaluated as a covariate in statistical analyses. Blood was stored on ice until processing within 6 h of collection. Penguins were weighed with spring scales and bill length and depth measured with digital calipers. Adult sex was determined using a discriminant function based on morphometrics (Bertellotti *et al.*, 2002). Adult females and males were in similar proportions between the tourist-visited and control sites within each colony (Supplementary data). *In vivo* cellular immune responses were only assessed for chicks because recapture after 24 h, as needed for this assay, could not be guaranteed for adults. Immediately after sampling, both chicks and the sampled adult were returned to their nest.

### Immune function

#### Leucocyte profile

Leucocyte counts provide information on the health-status and immunity of individuals (Campbell, 1995) and are considered important indicators in wild animals (Beldomenico *et al.*, 2008). Leucocyte profiles were obtained by scanning thin blood smears under a light microscope. Total leucocyte counts were estimated as the number of leucocytes per 10 visual fields at 400× (D’Amico *et al.*, 2014). The proportion of each leucocyte type (i.e. lymphocytes, heterophils, eosinophils, monocytes, and basophils) was obtained from a sample of 100 leucocytes under 1 000x magnification (oil immersion) (Campbell, 1995). Total counts for each leucocyte type were estimated by multiplying the total leucocyte count by the respective proportion. The heterophil/lymphocyte ratio (H/L ratio), an index of stress in vertebrates (Davis *et al.*, 2008), was calculated from the corresponding leucocyte proportions.

#### Bactericidal capacity of plasma

Several humoral innate components present in plasma interact to kill bacteria, including lysozymes and complement proteins (Roitt *et al.*, 1998). Bactericidal capacity of plasma constitutes a functionally relevant measure of immunity with increasing use in ecoimmunological studies (Matson *et al.*, 2006; Palacios *et al.*, 2011). We followed the protocol by Matson *et al.* (2006) with modifications for use in penguins. *Escherichia coli* (ATCC 8739) were suspended in sterile phosphate-buffered saline (PBS) to obtain a working solution containing 200–300 colony-forming bacteria per 10 μl. Plasma samples were diluted 1:10 with sterile PBS. Sample reactions consisted of 10 μl of bacterial solution and 90 μl of diluted plasma and were incubated for 8 min at 41°C. Three control reactions (10 μl of bacterial solution and 90 μl PBS) were plated before, midway, and after plating the sample reactions. All reactions were plated in duplicate using 50 μl aliquots on 4% tryptic soy agar and incubated at room temperature (~25°C) overnight until bacterial colonies were large enough to be easily observed. The number of bacterial colonies on each plate was then counted and the percentage of bacteria killed calculated.

#### Bacterial agglutination

Agglutination of foreign particles is mediated by plasma agglutinins such as natural antibodies. Natural antibodies recognize a broad array of pathogens (e.g. bacteria, viruses) and are involved in early resistance against infections (Ochsenbein and Zinkernagel, 2000). Agglutination of *E. coli* (ATCC 8739) was measured following a protocol that we had previously adapted for use in penguins (D’Amico *et al.*, 2014). Briefly, bacteria were grown in tryptic soy broth and fixed in 1% formalin overnight for 16–18 h at 4°C. Fixed bacteria were washed three times with PBS and adjusted to a concentration of ~1 ×10^9^ bacteria/ml. Plasma samples (20 μl) were added to the first column of a 96-well plate and serially diluted 2-fold with PBS. A negative control (PBS) was included in each plate and 20 μl of fixed bacteria were added to all wells. Plates were vortexed and incubated at room temperature (~25°C) overnight for 16–20 h. Agglutination titers were determined as −log2 of the highest dilution showing bacterial agglutination.

#### *In vivo* cellular immune response

The inflammatory response is an integral part of the defence against invading microorganisms (Roitt *et al.*, 1998). The local inflammatory response triggered by the subcutaneous injection of phytohemagglutinin (PHA) integrates the function of several leucocytes, including both innate (heterophils, basophils) and acquired (lymphocytes) components (Martin *et al.*, 2006). The skin-swelling response to the injection of PHA was performed following the protocol by Bertellotti *et al.* (2016). Briefly, chicks were injected with 0.1 ml of a 2-mg/ml solution of PHA (Sigma, L2646) in sterile PBS at a marked site on the interdigital membrane of the foot. The thickness of the foot web was measured with a digital thickness gauge (Mitutoyo, model PK-0505) with an accuracy of 0.01 mm at the injection site just before and 24 h after injection. The average of three thickness measures was considered at each time. The resulting swelling was calculated as the difference between the initial and final measurements.

### Blood biochemistry and haematocrit

Blood biochemical parameters contribute to the assessment of general body condition and nutritional status of birds (Brown, 1996; D’Amico *et al.*, 2017). A drop of whole blood was used to determine glucose level (mg/dl) using a portable devise (Accu-Check Performa, Roche). Total protein level (g/dl) in plasma was measured using a portable refractometer (Arcano) following manufacturer instructions. A heparinized microcapillary tube was filled with whole blood, centrifuged for 10 min, and haematocrit was measured as the percentage of packed red blood cells in the total blood volume. Haematocrit is considered a physiological index of general condition that can serve as an estimate of aerobic capacity (Fair *et al.*, 2007). Haematocrit data are only available for individuals sampled at Punta Tombo.

### Statistical analyses

Data for the two colonies were analyzed separately because the main effect of interest was that between tourist-visited and non-visited individuals within each colony and potential year effects, inter-assay variation, and age differences of chicks precluded suitable comparisons between colonies. The effect of gender on adult physiological parameters and response to tourism was evaluated using general linear models that included the fixed effects of sex, tourism (tourist-visited and non-visited), and their interaction. Physiological parameters of adults and chicks were then analyzed together using general linear mixed models to evaluate whether tourist visitation has a different effect on individuals from these two age classes. Models included the fixed effects of tourism and age (adults and chicks), the interaction between these two factors, and nest as a random effect. An exception was the model for the PHA response that was only measured in chicks. Body mass and condition (residuals of the regression of body mass on structural size) nested within each age group were alternatively evaluated as potential covariates. Similarly, sampling date and time, sampling order (order in which individuals were sampled within each area on each sampling date), and time elapsed between initial nest disturbance and end of blood collection were tested as a covariates in all models. Covariates were removed from the final models unless significant. Significance is reported at the *P* < 0.05 level; however, we also discuss results showing marginal significance (0.05 < *P* < 0.1) following recommendations for studies in conservation science (Fidler *et al.*, 2006; Field *et al.*, 2004). Effect sizes of statistically significant and marginally significant tourism effects were estimated using Cohen's *d*, Glass's Δ, and Hedge's *g* (Supplementary information) and effect size ranges reported. Residual plots from all models were visually inspected for signs of non-normality, and variables were transformed when necessary: leucocyte counts were log_10_-transformed and bactericidal capacity was arcsine-square-root-transformed. Basophil counts were low and with a predominance of zeros, so they are shown in graphs but not statistically analyzed. Untransformed raw data are depicted in graphs for visual clarity. Sample sizes differ among parameters due to limited blood sample volume or, in a few instances, sample loss. All statistical analyses were performed using JMP Pro 10.0.0 (SAS Institute Inc 2012).

## Results

### Adult gender effects

Genders did not differ in most physiological parameters, with the following exceptions. Glucose levels were significantly higher in males than females in both colonies and total eosinophil counts were significantly higher in females than in males in San Lorenzo (Supplementary data). Interactions between sex and tourism were never significant (all *P* > 0.15).

### Age group and tourism effects

#### Colony at Punta Tombo

**Figure 3:**
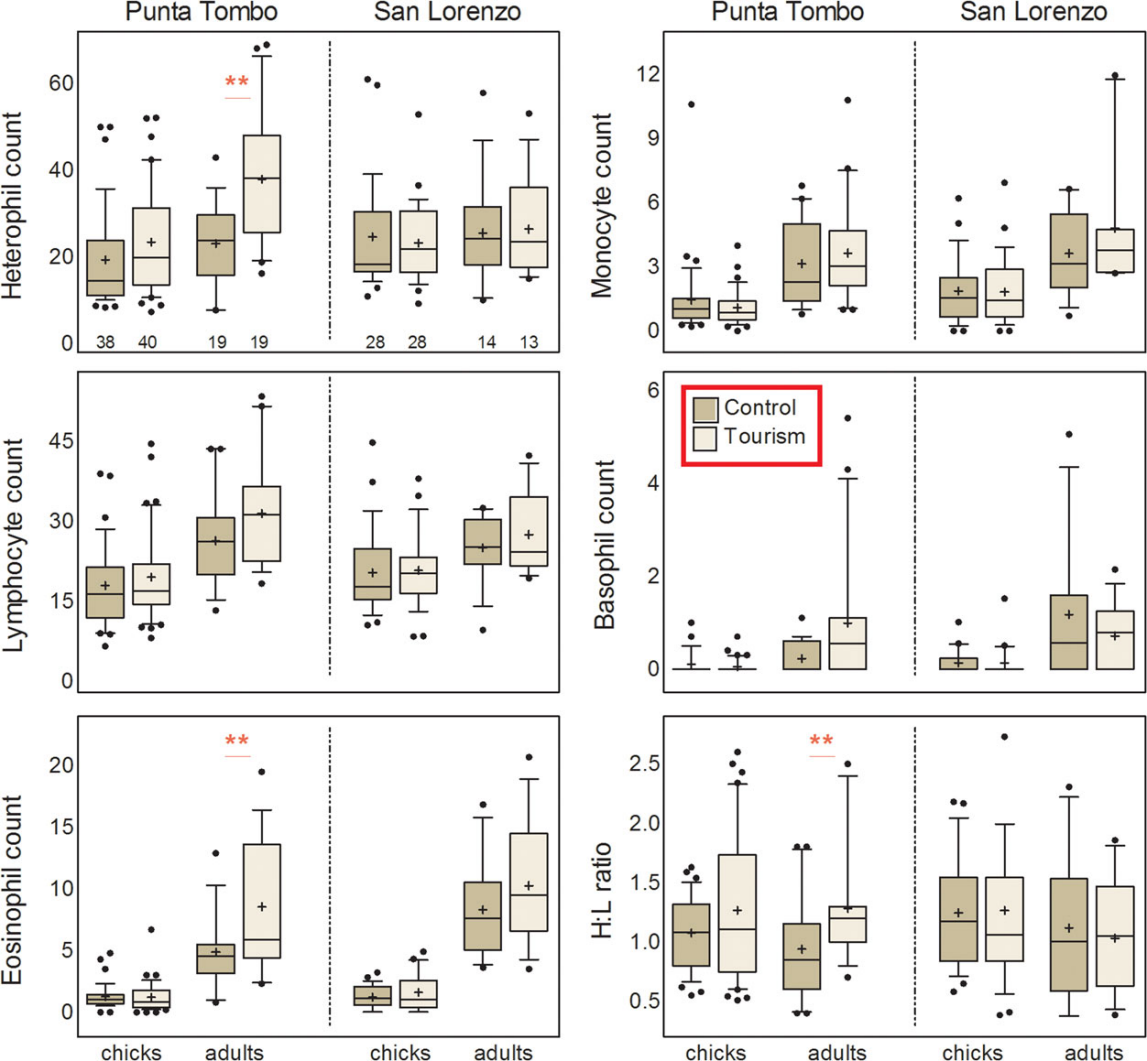
Box plots for leucocyte profile and H/L ratios of adult and chick Magellanic penguins at tourist-visited (Tourism) and non-visited (Control) sites at the two study colonies, Punta Tombo (left) and San Lorenzo (right). Box plots depict medians (horizontal lines inside boxes), means (crosses inside boxes), 25 and 75 percentiles (edges of boxes), 10 and 90 percentiles (whiskers) and outlying points included in the analyses (dots). Sample sizes are depicted below each box in the top left panel and are the same for all variables in this figure. Asterisks highlight the effect of site within each age group: ** = *P* < 0.05.

**Figure 4:**
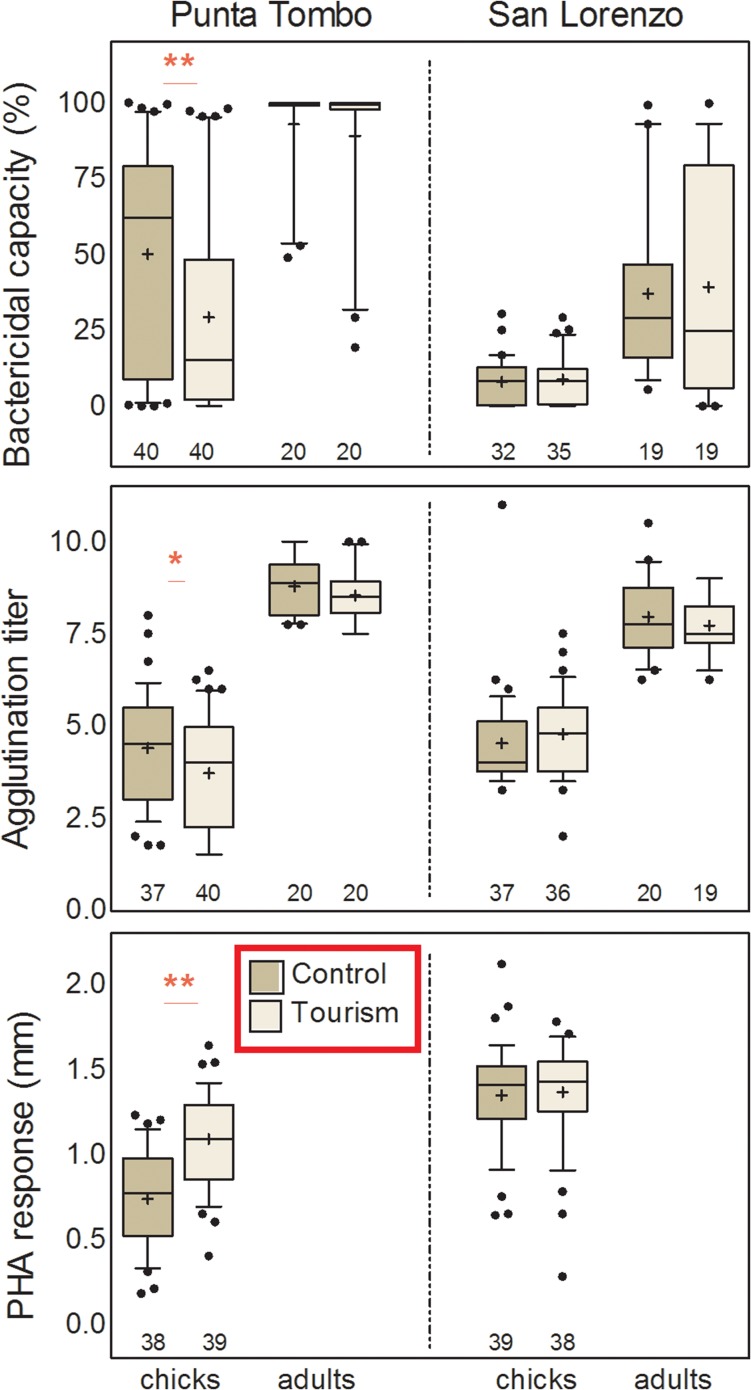
Box plots for immune parameters of adult and chick Magellanic penguins at tourist-visited (Tourism) and non-visited (Control) sites at the two study colonies, Punta Tombo (left) and San Lorenzo (right). Box plots depict medians (horizontal lines inside boxes), means (crosses inside boxes), 25 and 75 percentiles (edges of boxes), 10 and 90 percentiles (whiskers) and outlying points included in the analyses (dots). Sample sizes are depicted below each box. Asterisks highlight the effect of site within each age group: ** = *P* < 0.05, * = *P* < 0.1.

*Immune function*. All leucocyte counts were higher in adults than chicks (Table 2, Fig. 3). Heterophil and eosinophil counts showed a significant tourism by age interaction, with adults from the tourist-visited site, but not chicks, exhibiting elevated counts (Table 2, Fig. 3). Lymphocyte counts showed a marginal effect of tourism, being slightly higher in tourist-visited individuals (Table 2, Fig. 3). The H/L ratio, which did not differ between age groups, was higher in tourist-visited than control sites, with a marginal trend (*P* = 0.071) towards a tourism by age interaction (Table 2, Fig. 3). Separate analyses for each age showed that the effect of tourism on H/L ratios was only significant for adults (*F *= 6.79, *P* = 0.013). Both bactericidal capacity and bacterial agglutination titer were higher in adults than chicks (Table 2, Fig. 4). Bactericidal capacity also showed a marginal trend (*P* = 0.055) towards a tourism by age interaction and separate analyses for each age showed that the effect of tourism on bactericidal capacity was only significant for chicks (*F *= 6.71, *P* = 0.011) (Table 2, Fig. 4). Although bacterial agglutination titer did not show significant effects of tourism or tourism by age interaction in the general linear model (Table 2), visual inspection of the graph suggested lower titers for tourist-exposed than control chicks (Fig. 4) and a separate analysis by age showed this as a marginally significant effect (*F* = 3.72, *P* = 0.058). Finally, tourist-visited chicks exhibited higher inflammatory responses to PHA injection than did controls (Table 2, Fig. 4). The PHA response also showed a weak increase with body mass independently of site (Table 2, effect estimate: 2.9 ×10^−4^ ± 1.4 × 10^−4^ mm/g).

**Table 2:** General linear mixed models of physiological parameters of chick and adult Magellanic penguins in tourist-visited and non-visited (control) sites of two reproductive colonies in the coast of Patagonia

	Tourism			Age group			Tourism x Age group			Body mass		
**Punta Tombo**												
	*F*	*df*	*P*	*F*	*df*	*P*	*F*	*df*	*P*	*F*	*df*	*P*
Heterophils	10.90	1,41.5	**0.002**	20.85	1,74.2	**<0.0001**	4.75	1,74.2	**0.032**			
Lymphocytes	3.06	1,42.6	0.087	39.68	1,73.1	**<0.0001**	0.34	1,73.1	0.562			
Eosinophils	2.71	1,46.0	0.106	179.60	1,77.1	**<0.0001**	6.71	1,77.1	**0.012**			
Monocytes	0.01	1,39.3	0.926	61.78	1,70.1	**<0.0001**	1.72	1,70.1	0.194			
H/L ratio	5.95	1,41.5	**0.019**	0.64	1,74.8	0.427	3.35	1,74.8	0.071			
Bactericidal capacity	2.11	1,39.9	0.154	206.50	1,78.0	**<0.0001**	3.79	1,78.0	0.055			
Bacterial agglutination	2.24	1,42.5	0.142	423.52	1,75.9	**<0.0001**	0.95	1,75.9	0.331			
PHA response	17.07	1,38.5	**0.0002**	na			na			4.03	1,68.3	**0.049**
Glucose	26.12	1,38.9	**<0.0001**	19.59	1,84.1	**<0.0001**	20.39	1,67.1	**<0.0001**	5.82	2,84.4	**0.004**
Total protein	0.06	1,97.0	0.810	19.30	1,97.0	**<0.0001**	2.42	1,97.0	0.122			
Haematocrit	0.20	1,70.1	0.660	113.80	1,83.6	**<0.0001**	5.77	1,83.6	**0.019**			
**San Lorenzo**												
	*F*	*df*	*P*	*F*	*df*	*P*	*F*	*df*	*P*	*F*	*df*	*P*
Heterophils	0.03	1,31.9	0.867	0.70	1,54.2	0.406	0.23	1,54.2	0.633			
Lymphocytes	0.70	1,33.6	0.409	11.60	1,54.4	**0.001**	0.22	1,54.4	0.642			
Eosinophils	1.16	1,31.6	0.290	183.41	1,54.2	**<0.0001**	0.19	1,54.2	0.668			
Monocytes	0.72	1,32.9	0.400	30.54	1,54.1	**<0.0001**	1.08	1,54.1	0.303			
H/L ratio	0.30	1,30.0	0.587	2.67	1,52.9	0.108	0.11	1,52.9	0.737			
Bactericidal capacity	0.002	1,39.4	0.968	58.09	1,66.0	**<0.0001**	0.004	1,66.0	0.952			
Bacterial agglutination	2.23	1,42.5	0.142	423.52	1,75.9	**<0.0001**	0.95	1,75.9	0.332			
PHA response	0.07	1,37.1	0.795	na			na					
Glucose	0.23	1,53.3	0.630	8.70	1 111.8	**0.004**	0.83	1,82.9	0.364	9.02	2 104.3	**0.0002**
Total protein	0.05	1,40.1	0.831	331.20	1,71.8	**<0.0001**	5.40	1,71.8	**0.023**			

Abbreviations: na: not applicable, H/L: heterophil/lymphocyte, PHA: phytohemagglutinin. Significant effects are highlighted in bold.

**Figure 5:**
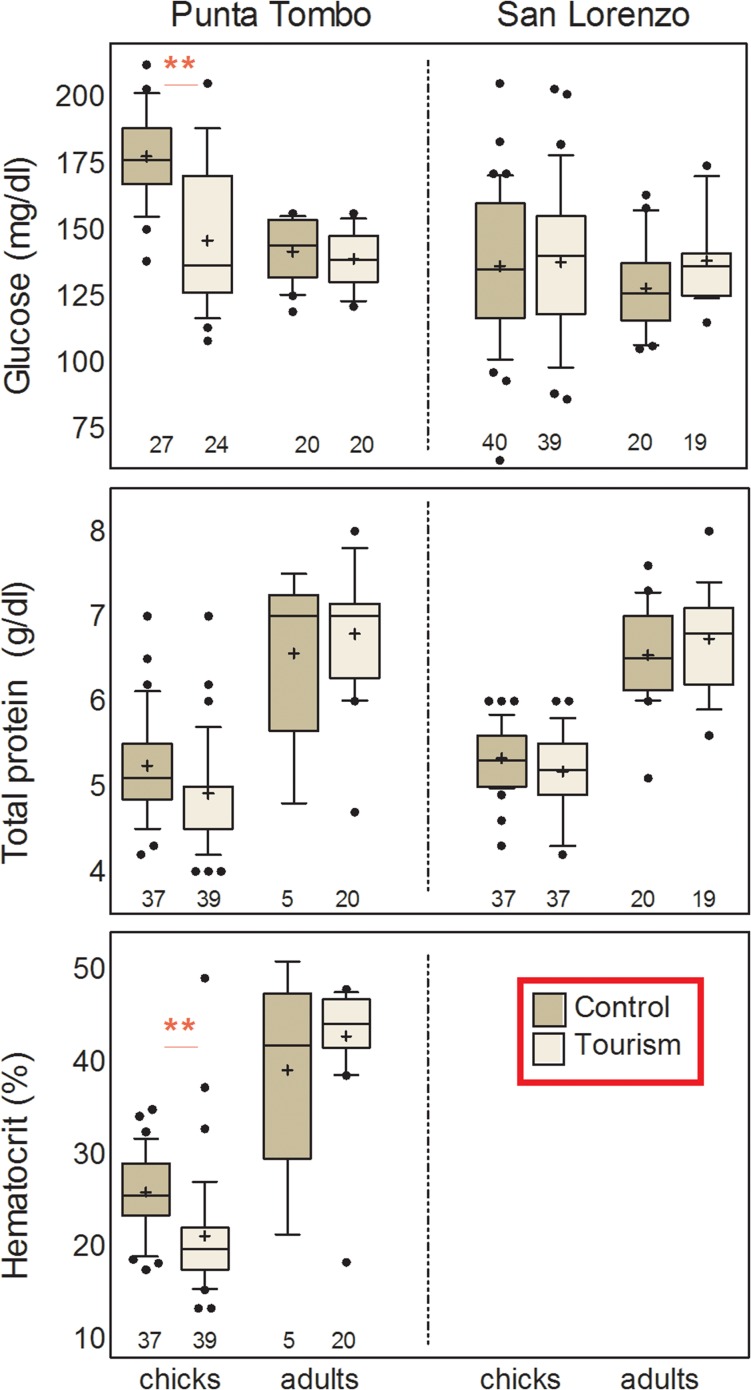
Box plots for haematological biochemical parameters and haematocrit of adult and chick Magellanic penguins at tourist-visited (Tourism) and non-visited (Control) sites at the two study colonies, Punta Tombo (left) and San Lorenzo (right). Box plots depict medians (horizontal lines inside boxes), means (crosses inside boxes), 25 and 75 percentiles (edges of boxes), 10 and 90 percentiles (whiskers) and outlying points included in the analyses (dots). Sample sizes are depicted below each box. Asterisks highlight the effect of site within each age group: ** = *P* < 0.05.

*Blood biochemistry and haematocrit*. Glucose levels showed a significant interaction between tourism and age, with tourist-visited chicks, but not adults, showing lower levels than non-visited individuals (Table 2, Fig. 5). In addition, glucose levels increased with body mass within each age (Table 2). Total protein levels only varied significantly with age, being higher in adults than chicks (Table 2, Fig. 5). Haematocrit was higher in adults than chicks and showed a significant tourism by age interaction, with tourist-visited chicks, but not adults, exhibiting reduced haematocrit compared to those of non-visited individuals (Table 2, Fig. 5).

#### Colony at San Lorenzo

*Immune function*. None of the immune parameters differed significantly between penguins at tourist-visited and control sites; neither were there significant interactions between tourism and age (Table 2, Figs 3 and 4). Lymphocyte, eosinophil, and monocyte counts were higher in adults than chicks, whereas heterophil counts and H/L ratio showed no age effect (Table 2, Fig. 3). Bactericidal capacity and bacterial agglutination were also higher in adults than chicks (Table 2, Fig. 4). PHA responses of chicks showed no effects of tourism or body mass (Table 2, Fig. 4).

*Blood biochemistry*. Glucose levels were significantly higher in adults than chicks, increased with body mass within each age group, but showed no effect of tourism or tourism by age interaction (Table 2, Fig. 5). Total protein levels were higher in adults than chicks and showed a significant tourism by age interaction, with tourist-visited penguins displaying a slightly greater difference in protein levels with age than controls (Table 2, Fig. 5).

## Discussion

Magellanic penguin adults and chicks exposed to tourism in the colony at Punta Tombo exhibited alterations in diverse physiological indices of immune and general-health condition, whereas those in the colony at San Lorenzo did not. These results highlight the power of a conservation physiology approach including a diverse panel of physiological indices for understanding anthropogenic effects on wild animals (Carey, 2005; Wikelski and Cooke, 2006; Madliger *et al.*, 2016) and are in line with the altered corticosterone-mediated stress responses of adult and chicks exposed to tourist visits at Punta Tombo (Walker *et al.*, 2005, 2006), but not at San Lorenzo (Villanueva *et al.*, 2012). Together, these results indicate clear and consistent physiological consequences of tourist activities on penguins at Punta Tombo, whereas penguins exposed to visitation at San Lorenzo do not seem to be affected. We next discuss the specific physiological changes documented at Punta Tombo (summarized in Table 3) before exploring the potential causes of the disparate patterns between colonies.

**Table 3:** Summary of immune and general-health physiological alterations of tourist-visited penguins relative to non-visited controls in the colony at Punta Tombo and their effect size ranges (see Statistics for details)

Parameter	Direction of effect	Effect size range
***Adults***		
Heterophil counts	Higher	1.14–1.26
Eosinophil counts	Higher	0.54–0.57
H/L ratio	Higher	0.80–0.88
***Chicks***		
Bactericidal capacity	Lower	0.58–0.59
Bacterial agglutination	Lower^a^	0.44–0.45
PHA response	Higher	1.19–1.20
Glucose levels	Lower	1.43–1.96
Haematocrit	Lower	0.84–1.1

^a^Marginally significant effect.

### Effects of tourism on penguin physiology at Punta Tombo, the declining and intense tourism colony

Tourist-visited adult penguins showed higher H/L ratios and heterophil and eosinophil counts than non-visited controls. Elevated H/L ratios are consistent with chronic stress in tourist-visited adults at Punta Tombo, and are in line with the depressed hypothalamo-pituitary-adrenal function documented at this same location more than a decade ago (Walker *et al.*, 2005). Elevated heterophil and eosinophil counts are generally associated with infections (Campbell, 1995; Thrall *et al.*, 2012). In particular, elevated eosinophils are found in individuals infected by gastrointestinal parasites and high prevalence and intensity of gastrointestinal helminths have been reported for Magellanic penguins (Diaz *et al.*, 2010). The elevated eosinophils of tourist-visited adults at Punta Tombo could thus reflect greater infection by endoparasites, a pattern of parasitism that has been reported for other tourist-exposed animals such as rock iguanas (Knapp *et al.*, 2013). Chronic stress is generally immunosuppressive (Martin, 2009), increasing the risk and severity of infections (Beldomenico and Begon, 2015). In this respect, it is interesting to note that humoral innate immune parameters were not affected by tourism in adults, suggesting that while certain immune functions could be depressed, others seem to be maintained despite the higher overall stress.

Chicks born to, and reared by, these stressed adults, and exposed to tourism themselves, showed alterations in several physiological functions with respect to control chicks. In terms of immunity, tourist-exposed chicks showed greater skin-swelling responses to PHA and lower bactericidal capacity and agglutination titers. Higher PHA responses are usually interpreted as stronger cellular immunity, as several studies have found positive effects of nutrition and condition (or body mass) on this response (e.g. Alonso-Alvarez and Tella, 2001; Tella *et al.*, 2001; this study for chicks in Punta Tombo) and higher responses have been linked to greater survival (Moller and Saino, 2004). Nevertheless, other studies have found increased PHA responses in individuals infested by haematophagous ectoparasites (e.g. Dusbabek *et al.*, 1988; Gwinner *et al.*, 2000), interpreting them as the result of prior sensitization of the immune system to antigenic products introduced when parasites feed on their hosts. We suspect that the latter is likely the case here, as we noticed higher flea prevalence in tourist-visited than in control chicks at Punta Tombo, a pattern that we have now confirmed (Supplementary data). In addition, tourist-visited chicks showed lower bactericidal capacity and a trend towards lower bacterial agglutination. This points to a depressed constitutive humoral innate immunity, involving important effectors such as natural antibodies, the complement system, and lysozyme. Given that young individuals tend to rely more on innate than acquired responses (Apanius *et al.*, 1998; Palacios *et al.*, 2009), reduced or altered innate immunity of chicks could have negative fitness effects and warrants further study.

Other physiological alterations of tourist-exposed chicks at Punta Tombo included lower blood glucose levels and haematocrits. These parameters provide information regarding general body condition and nutritional status. In accordance, glucose levels increased with increasing body mass within each age group in both penguin colonies. Poor nutrition and unpredictable food availability can lead to lower haematocrits (Acquarone *et al.*, 2002; Cucco *et al.*, 2002). Thus, the lower glucose levels and haematocrits of tourist-visited chicks could result from stressed parents at tourist-visited sites not being able to deliver enough food (in quantity or quality) and/or chicks not being able to assimilate food as efficiently. In addition, low haematocrits can also be caused by hematofagous ectoparasites (e.g. Fair *et al.*, 2007; Gwinner *et al.*, 2000), which is consistent with the higher flea prevalence observed in tourist-visited chicks. Overall, these results suggest that at Punta Tombo, tourist visitation leads to lower general-health and immunological condition of chicks and further reinforce the notion that their higher PHA responses might be more related to hypersensitization by ectoparasites than to stronger cellular immunity.

### Effect of adult gender and differences between mature and immature penguins

Many studies on diverse physiological effects of ecotourism on wildlife have focused primarily on adult individuals (e.g. Amo *et al.*, 2006; Barbosa *et al.*, 2013; French *et al.*, 2010; Semeniuk *et al.*, 2009; Thiel *et al.*, 2008). Among studies including both adult genders, different effects of tourism on male versus female physiology have been reported in some (e.g. Knapp *et al.*, 2013; Thiel *et al.*, 2008), but not other cases (e.g. Amo *et al.*, 2006; French *et al.*, 2010). Magellanic penguins seem to fall in the latter category, as genders showed very few differences in their immune and general-health parameters and no differences in their response to tourist visitation (i.e. sex by tourism interaction was never significant). On the other hand, the present study emphasizes the importance of assessment of both adult and immature individuals to more fully understand the impact of anthropogenic activities on wildlife. Immature or developing individuals have specific physiological requirements and challenges (i.e. growth and development instead of reproduction), and might therefore show different sensitivity to stressors compared to adults. Walker *et al.* (2005, 2006) demonstrated opposite effects of tourist visitation on the stress response of Magellanic penguin adults versus chicks. Our results expand the latter by demonstrating that different physiological aspects related to general-health and immune function are most affected in adults versus chicks exposed to tourist visitation at Punta Tombo (Table 3).

### Why does tourism show an effect at Punta Tombo but not at San Lorenzo?

Despite tourist visitation at San Lorenzo, no immunological or general-health effects were detected on tourist-exposed penguins in that colony. A more recent history of tourist activity and less intense tourism practices at San Lorenzo compared to Punta Tombo could in principle have contributed to the observed differences, as it has been suggested to explain the disparate effects of tourism on stress responses (Villanueva *et al.*, 2012). Nevertheless, between-year differences could also have contributed to the observed patterns in our study. In particular, 2015 was an El Niño year whereas 2013 was not. Thus, harsher climatic factors could have resulted in an extra stressor for individuals sampled at Punta Tombo in 2015, making the effect of tourism more pronounced. Yet, another important difference between the two colonies is that the penguin breeding population at Punta Tombo is in decline, whereas the one at San Lorenzo is increasing (Pozzi *et al.*, 2015). Given that the area opened to tourists is a minor part of the total area within each colony, it is unlikely that tourist activity *per se* is driving these opposing population trends. Rather, these trends are likely related to density-dependent effects in older, larger colonies such as Punta Tombo coupled with lower and less predictable food availability in southern areas such as where Punta Tombo is located (Pozzi *et al.*, 2015). Thus, penguins at the declining colony at Punta Tombo, already showing lower reproductive success and recruitment (Pozzi *et al.*, 2015), could be more susceptible to additional stressors than penguins at the growing colony at San Lorenzo, further contributing to the different physiological responses to tourism in the two colonies. Additional studies would be required to determine the relative contributions of the above factors in explaining the different patterns observed between the two reproductive colonies.

### Conservation and management implications

Numerous wildlife populations are currently the focus of tourist visitation and more will become exposed to this activity in coming years. The conservation status of Magellanic penguins has changed from ‘Lower Risk/least concern (LR/lp)’ in 1994 to ‘Near Threatened (NT)’ in 2004 (Birdlife International, 2017). The species is vulnerable to oil pollution, commercial fisheries, and climate change (Boersma, 2008; Boersma and Rebstock, 2014). Our study supports the notion that tourist visitation is an additional factor that needs to be considered and that could interact with other environmental stressors in driving dynamics in this and other wildlife populations (Bertellotti *et al.*, 2013). Furthermore, the different responses found between adults and chicks and between the two Magellanic penguin colonies emphasize the importance of site-specific knowledge for informing and developing sustainable ecotourism management guidelines (Seddon and Ellenberg, 2008). Given the growing ecotourist demand, more wildlife populations might become exposed to this activity in coming years. Although specific guidelines should be evaluated and adapted for each case, some general measures could potentially help minimize effects of tourism on the most sensitive wildlife populations (e.g. Ellenberg, 2017; Samia *et al.*, 2017; Semeniuk *et al.*, 2009). For instance, in the case of penguins: (1) reduce the time individuals are exposed to visitors by reducing visitor numbers and/or visiting hours, or by improving circulation in walking paths to avoid overcrowding, (2) increase the minimum visitor approach distance to individuals and nests and/or use hides and covered trenches for closer range watching, (3) improve visitor education and awareness to avoid close interactions with the animals, including taking close range photographs such as selfies, and (4) continuously monitor populations and strengthen collaboration among researchers, tourism managers, and conservation authorities. The present study reemphasizes how the use of a diverse physiological toolkit within a conservation physiology approach can provide valuable information for a better and more complete comprehension of anthropogenic effects on wild animals in our changing world. Further studies using such tools are warranted to help develop and evaluate specific adaptive management plans.

## Supplementary Material

Supplementary Data
