# Supplementary material for: Ecotourism effects on health and immunity of Magellanic penguins at two reproductive colonies with disparate touristic regimes and population trends
Source: Conserv Physiol. 2018 Nov 13;6(1):coy060. doi: 10.1093/conphys/coy060 (PMC6233856; doi:10.1093/conphys/coy060)
Supplement: Supplementary Data [file coy060_supporting_information_revised.pdf]

Supporting Information

Ecotourism impacts on health and immunity of Magellanic penguins at reproductive colonies with disparate touristic regimes and population trends.

- ***Additional information on the two Magellanic penguin colonies studied***

Both colonies are opened to visitors during the reproductive season (September to April). In both colonies tourists are only authorized to walk inside the delineated walking trails in the visited area of each colony, which covers a very small percentage (less than 1%) of the total colony area. In both colonies tourists can get close to penguins (within 1 m) when these stand or nest close to the walking trails and/or when they cross the trails to get back and forth from their nests to the seashore.

Punta Tombo

The colony at Punta Tombo covers an area of approximately 400 ha. Punta Tombo is opened to the public between 8:00 and 20:00 hs daily and has an almost permanent mode of visitation, with main trails of double circulation (i.e., people walk along the same path on the way in and out of the visiting area) that result in a practically permanent presence of visitors in the walking trails. Trails (ca. 2 km long) are at ground level and marked in the gravel in some parts of the visited-area, whereas in other parts elevated board-walks have been built under which penguins can walk or take shelter. Visitors can be part of guided groups (10-60 tourists) or can also visit independently without a guide. Punta Tombo has a visitor center and park rangers who welcome tourists, talk about expected tourist behavioral rules, and patrol the trails. For further details, including landscape features, see also: Yorio and Boersma (1992), Rebstock et al. (2016); full references in main text

San Lorenzo

The colony at estancia San Lorenzo covers an area of approximately 100-150 ha (area has been expanding with the growth of the population). Visiting hours during the reproductive season are between 11:00 and 18:00. Visits are usually conducted in discrete, guided groups of no more than 25 people, who walk along a one-way circular path (loop of ca. 800 m long). Trails are mainly at ground level and

marked on the natural gravel floors. There are no park rangers at San Lorenzo. For further details see also: Villanueva et al. (2012), Villanueva et al. (2014); full references in main text

- ***Genders were in similar proportions between tourist-visited and control adults at both colonies***

	**Punta Tombo**		**San Lorenzo**	
	Females	Males	Females	Males
**Tourist-visited adults**	7	13	8	12
**Control adults**	7	13	10	10

- ***Adult gender effects on physiological parameters***

• Glucose levels (mg/dl) were lower in adult females (F) than males (M) in both reproductive colonies.
Punta Tombo (mean ± SE): F= 132.14 ± 2.52, M = 144.62 ± 1.85, $F_{1,1}$ = 15.97, P = 0.0003, n = 38
San Lorenzo: F = 125.46 ± 3.47, M = 139.35 ± 1.85, $F_{1,1}$ = 8.68, P = 0.006, n = 39

• Adult females in San Lorenzo showed higher total eosinophil counts than males.
F = 11.07 ± 1.12, M = 7.32 ± 1.15, $F_{1,1}$ = 5.4, P = 0.03, n = 27

- ***Higher flea prevalence in tourist-visited than in control chicks at Punta Tombo in 2016***

	**Fleas absent**	**Fleas present**	**Prevalence**
**Tourist-visited chicks**	11	22	0.66
**Control chicks**	29	2	0.06

ChiSquare: 27.839, P < 0.0001*

- ***Effect sizes***

Effect sizes were calculated using the online calculators by Ellis, P.D. (2009):

"Effect size calculators," website https://www.polyu.edu.hk/mm/effectsizefaqs/calculator/calculator.html accessed on September 2018.

The three better-known indexes are reported. For details visit the above website or the references original below.

Cohen, J. (1988), *Statistical Power Analysis for the Behavioral Sciences*. Hillsdale: Lawrence Erlbaum.

Glass, G.V., B. McGaw, and M.L. Smith (1981), Meta-Analysis in Social Research. Sage: Beverly Hills.

Hedges, L.V. and I. Olkin (1985), *Statistical Methods for Meta-Analysis*. London: Academic Press.

Parameter	Treatment	n	mean	SD	Effects sizes	
CHICKS						
**PHA response**	Tourism	39	1.09	0.29	**Cohen´s d**	1.2
	Control	38	0.74	0.29	**Glass's Δ**	1.2
					**Hedges g**	1.19
**Bactericidal capacity**	Tourism	40	0.51	0.46	**Cohen´s d**	0.58
(angular transform)	Control	40	0.78	0.47	**Glass's Δ**	0.59
					**Hedges g**	0.58
**Glucose**	Tourism	24	145.92	26.89	**Cohen´s d**	1.43
	Control	27	177.56	16.13	**Glass's Δ**	1.96
					**Hedges g**	1.43
**Hematocrit**	Tourism	39	21.13	6.52	**Cohen´s d**	0.86
	Control	37	25.87	4.31	**Glass's Δ**	1.1
					**Hedges g**	0.84
**Agglutination titer**	Tourism	40	3.71	1.56	**Cohen´s d**	0.44
	Control	37	4.39	1.52	**Glass's Δ**	0.45
					**Hedges g**	0.44
ADULTS						
**H/L ratio**	Tourism	19	0.08	0.16	**Cohen´s d**	0.88
(log_{10} transform)	Control	19	-0.08	0.20	**Glass's Δ**	0.8
					**Hedges g**	0.86
**Heterophil counts**	Tourism	19	1.56	0.17	**Cohen´s d**	1.26
(log_{10} transform)	Control	19	1.32	0.21	**Glass's Δ**	1.14
					**Hedges g**	1.23
**Eosinophil counts**	Tourism	19	0.91	0.25	**Cohen´s d**	0.57
(log_{10} transform)	Control	19	0.76	0.28	**Glass's Δ**	0.54
					**Hedges g**	0.55
